# Clinical and genetic characteristics of PSTPIP1-associated myeloid-related proteinemia inflammatory syndrome

**DOI:** 10.1186/s12969-021-00636-9

**Published:** 2021-10-07

**Authors:** Dan Zhang, Gaixiu Su, Yan Liu, Jianming Lai

**Affiliations:** 1 Department of Rheumatology, Capital institute of pediatrics, 2 yabao road, chaoyang district, Beijing, China; 2 Department of Rheumatology, Dalian municipal Women and Children’s Medical Center, No.1 and No.3, Sports newtown planning road 1,Ganjingzi district, Dalian City, Liaoning China

**Keywords:** PSTPIP1-associated myeloid-related proteinemia inflammatory syndrome, Tumour necrosis factor antagonist

## Abstract

**Objective:**

To summarise the clinical and genetic characteristics of three children with PSTPIP1-associated myeloid-related proteinemia inflammatory (PAMI) syndrome.

**Methods:**

This study retrospectively analysed the clinical and genetic data of three children with PAMI syndrome in our hospital between April 2018 and January 2020.

**Results:**

One male and two female children were 6 years and 5 months, 8 years and 7 months, and 13 years and 3 months of age. All three patients had a recurrent blood trilineage hypoplasia and splenomegaly. Patient 1 had pyoderma gangrenosum, and Ludwig’s angina. Patient 2 had pyogenic arthritis, and pyoderma gangrenosum. Patient 3 had hepatomegaly, pyogenic arthritis, and pulmonary hypertension. Laboratory tests revealed that all three children had elevated C-reactive protein (CRP) and erythrocyte sedimentation rate (ESR). Patient 1: C-antineutrophilic cytoplasmic antibodies(c-ANCA), positive; antiglobulin test (Coombs), positive. Patient 2: blood zinc, 4.38 mg/L (elevated). Patient 3: Antinuclear antibodies (ANA), 1:100, β2 glycoprotein I, positive; Coombs test, positive; RF, 28.3 U/ml (elevated); C3, 0.77 g/L (decreased). Genetic testing showed that all 3 patients had PSTPIP1 c.748G > A (p.E250K) spontaneous heterozygous mutations, suggesting the diagnosis of PAMI syndrome. Patient 1 was treated with a combination of methylprednisolone and cyclosporine for 8 months. The patient did not develop new skin lesions. The blood count showed mild neutropenia. The spleen was considerably retracted and the CRP became normal. Patient 2 was treated with etanercept and methylprednisolone. The patient had no further arthralgias and pyoderma gangrenosum showed improvement. The spleen was smaller than before. White blood cells were shown to be approximately 2–3 × 10^9^/L. The haematocrit, platelets, CRP, and AESR were normal. Patient 3 was treated with methylprednisolone, methotrexate, and infliximab 4 times. The patient’s joint symptoms disappeared gradually and the liver retracted markedly. The pulmonary artery pressure returned to normal. Moreover, Coombs test result was negative. CRP and AESR were lower than before.

**Conclusion:**

PAMI syndrome can manifest as pyogenic arthritis, pyoderma gangrenosum, acne, and trilineage hypoplasia, as well as autoimmune diseases. Glucocorticoid and immunosuppressive therapy are partially effective and cytokine antagonists can be used in refractory cases. Whole-exome genetic testing is helpful to confirm diagnosis.

## Keypoint

- PAMI syndrome can be manifested as an autoinflammatory disease. It can also show features of autoimmune diseases.
- PAMI syndrome is highlighted by intractable decline of leukocyte, which is difficult to treat. Cyclosporine may be effect for leukopenia. TNFα antagonists are effective against pyoderma gangrene and pyogenic arthritis, Steriod plays an important role in the treatment of this disease.
- Clinical and genetic characteristics of PSTPIP1-associated myeloid-related proteinemia inflammatory syndrome.

## Background

PSTPIP1-associated myeloid-related proteinemia inflammatory syndrome (PAMI syndrome), is a rare autoinflammatory disease caused by pathogenic variants of the PSTPIP1 gene. PAMI syndrome is characterised by chronic systemic inflammation, pyogenic arthritis, hepatosplenomegaly, growth retardation, and may show elevated serum zinc levels and elevated myeloid-related protein (MRP)-8/14 complexes. The pathophysiological mechanisms of PAMI syndrome are not fully understood. However, the specific mutations in PSTPIP1 (p.E250K and p.E257K) are thought to be caused by significantly increased binding of immunomodulatory protein pyrin due to the charge reversal of ɣ domain. Steriod, immunosuppressants, and biologics have all been reported to be effective for this disease. Clinical reports of this disease have increased recently due to widespread use of whole-exome genetic testing. Three children were diagnosed with PAMI syndrome in our hospital, all with early age of onset. One patient had pancytopenia and rash, one had pancytopenia and pyogenic arthritis, and another had arthritis, leukopenia, autoimmune haemolytic anaemia, positive ANA, positive β2 glycoprotein, and declined complement. Tumour necrosis factor antagonists were used in two of the three cases, with remarkable clinical efficacy and few side effects. We analysed the clinical data and genetic test results of these three cases to enhance our clinical understanding, diagnosis accuracy, and treatment efficacy of this disease.

## Methods

We retrospectively analysed clinical data and genetic testing results of three children with PAMI syndrome diagnosed at the Department of Rheumatology and Immunology, Capital Institute of Pediatrics, between April 2018 and January 2020. One patient was male, and two patients were female. We collected their medical history, including age of onset, age at diagnosis, medical history, physical examination, laboratory tests, and whole-exome gene testing. Informed consent was obtained from the patients’ parents, who signed a written document.

## Results

The clinical manifestations of the three children are summarised in Table 1, and the laboratory test data are summarised in Table 2.

**Table 1:** Clinical manifestations of three patients

	Patient 1	Patient 2	Patient 3
Fever	+	–	+
Failure to thrive	–	+	–
Splenomegaly	+	+	+
Pyogenic arthritis	–	+	+
Pyoderma gangrenosum	+	+	–
Acne	–	–	–
Pulmonary arterial hypertension	–	–	+

**Table 2:** Laboratory examination of three patients

	patient1	patient2	patient3
WBC(×10^9^/L)	1.68	1.87	1.75
HB(g/L)	95	53	98
PLT(×10^9^/L)	63	125	45
CRP (mg/L)	111	69	139
AESR (mm/60 min)	N	140	91
serumzinc (mg/L)	N	4.38	N
calprotectin (ug/L)	N	72.4	**N**
ANA	–	–	1:100
ANCA	+	–	–
Coombs	+	+	+
RF	–	–	+
β2GPI	N	–	+
C3(g/L)	Normal	Normal	C3 0.77 g/L

Clinical diagnosis and genetic test identified c.748G > A variant in PSTPIP1 gene of three patients. The c.748G > A (nucleotide 748 in the coding region changed from guanine to adenine) variant causes the p.E250K mutation (amino acid 250 changed from glutamic acid to lysine), which is a missense mutation. The bioinformatics protein function software SIFT, PolyPhen_2, and REVEL predicted it to be deleterious, deleterious, and benign, respectively.

The PSPPIP1 gene follows an autosomal dominant mode of inheritance and was identified by Sanger in three family lines, all of which were identified as spontaneous mutations in probands, thus demonstrating consistency within their family.

### Patient 1

**Fig. 1:**
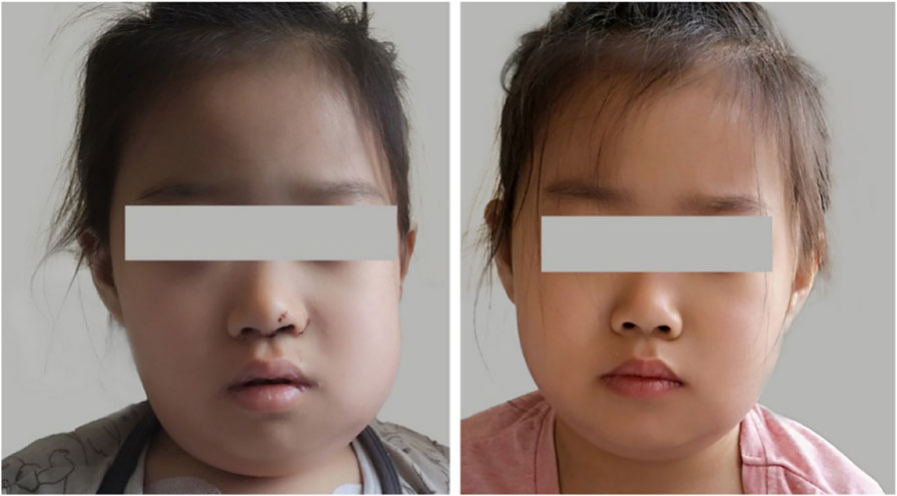
The face of the patient 1(Comparison before and after treatment)

**Fig. 2:**
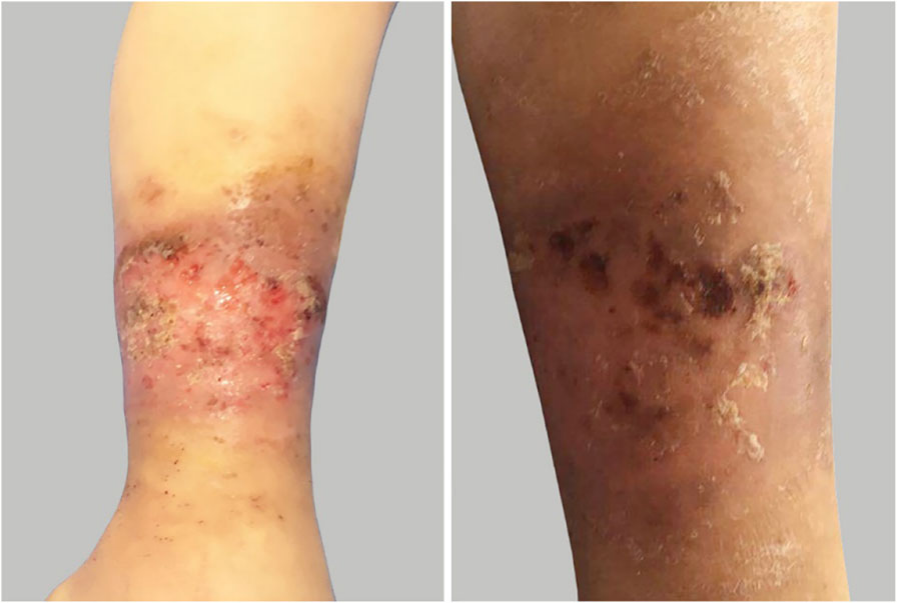
The leg of the patient 1(Comparison before and after treatment)

**Fig. 3:**
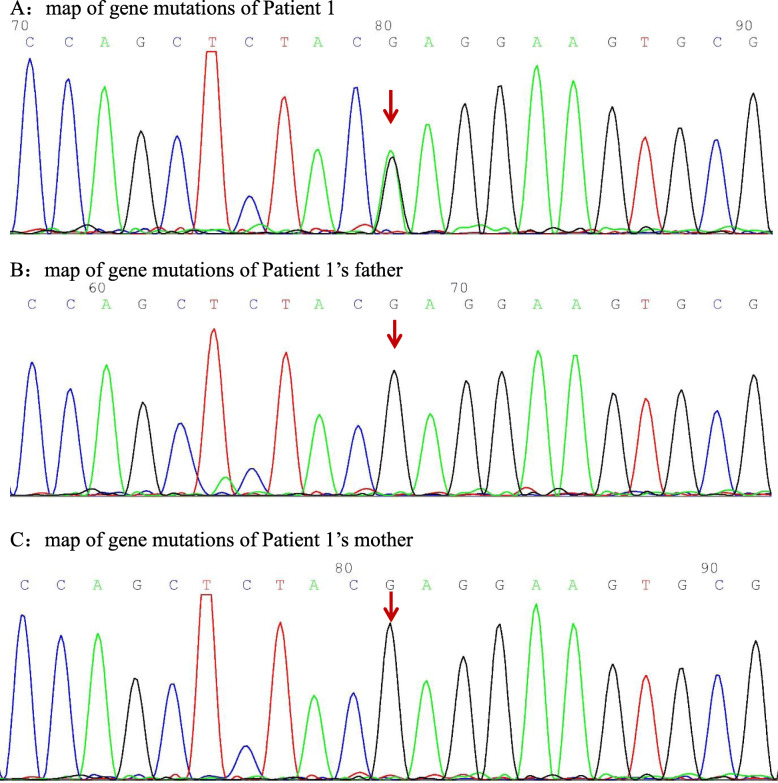
**A**:map of gene mutations of Patient 1. **B**:map of gene mutations of Patient 1’s father. **C**:map of gene mutations of Patient 1’s mother

Three years ago, an 8-year-old female patient was admitted to the hospital with fever, systemic rash, and trilineage hypoplasia. The rash was round, yellow-brown patches on the trunk and proximal extremities, with bran-like scales on the surface, accompanied by itching. Her spleen was hard and enlarged (10 cm) below ribs. Intravenous human immunoglobulin therapy 2 g/kg was ineffective. Complete blood count monitoring revealed: reduced white blood cell (WBC), 1.68 × 10^9^/L; reduced neutrophil, 0.66 × 10^9^/L; reduced haemoglobin (Hb), 95 g/L; reduced red blood cell (RBC), 2.4 × 10^12^/L; reduced reticulocyte, 0.034; reduced platelet (PLT), 63 × 10^9^/L; and elevated C-reactive protein (CRP), up to 111 mg/L. Anti-neutrophil cytoplasmic antibody (ANCA) tested was positive; antiglobulin test (Coombs) was positive, and bone marrow aspiration suggested trilineage hypoplasia. The patient was considered to have Ludwig’s angina two years ago when she had a relapsing fever, left swelling and painful jaw (Fig. 1), and skin ulceration (Fig. 2). The culture obtained from the exudates was positive for *Staphylococcus aureus*. Moreover, the left jaw was swollen and painful with progressive dysphagia. The whole-exome gene testing was completed and suggested a spontaneous PSTPIP1 c.748G > A (p.E250K) (see Fig. 3 for gene mutation profile). Therefore, she was diagnosed with PAMI syndrome. Oral Methylprednisolone (1 mg/kg/d) and cyclosporine (4 mg/kg/d) were administered, and the left mandibular swelling gradually disappeared (Fig. 1). The treatment was followed up for two years. Methylprednisolone and cyclosporine was reduced to 3 mg/d and 75 mg/d, respectively. Only her old skin lesion existed persistively (Fig. 2). Her blood cell count and CRP levels returned to normal. Her splenomegaly recovered.

### Patient 2

**Fig. 4:**
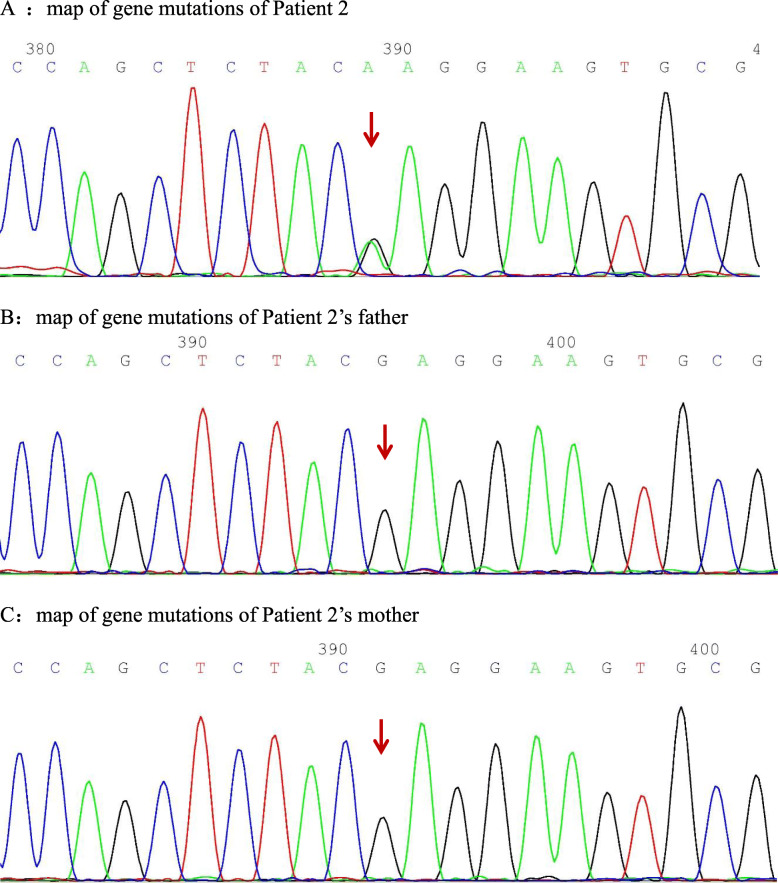
**A**:map of gene mutations of Patient 2. **B**:map of gene mutations of Patient 2’s father. **C**:map of gene mutations of Patient 2’s mother

**Fig. 5:**
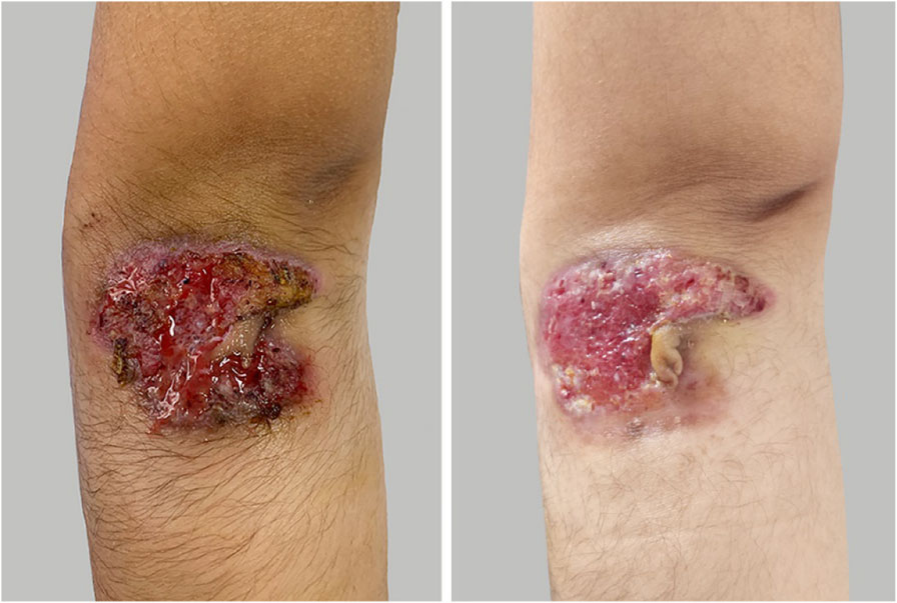
The elbow of the patient 2 (Comparison before and after treatment)

**Fig. 6:**
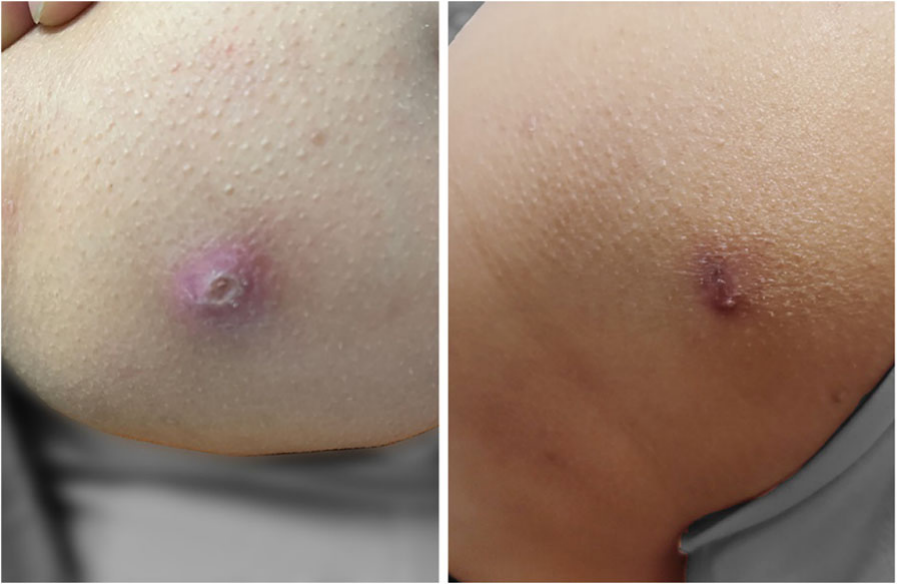
The buttock of the patient 2 (Comparison before and after treatment)

Eight years ago, a 10-year-old female patient underwent a bone marrow aspiration to investigate growth retardation and splenomegaly, which indicated trilineage hypoplasia. Symptomatic blood transfusion treatment was given. However, the patient’s condition did not improve. Five years ago, she developed pain in the right hip joint with limited mobility, which was considered as a hip abscess. The joint cavity was punctured and drained, and the pus was cultured, which was negative. The joint symptoms improved following the treatment of antibiotics, component blood transfusion, and intravenous human immunoglobulin. Blood investigation showed: WBC 1.87–2.66 × 10^9^/L; reduced neutrophil, 0.42 × 10^9^/L; Hb 53–80 g/L; reduced RBC 2.32–2.45 × 10^12^/L; PLT 125–152 × 10^9^/L; CRP 8–69 mg/L; AESR 19–140 mm/60 min; SF 520.18 ng/ml; and serum zinc 4.38 mg/L. Whole-exome genetic testing suggested a spontaneous PSTPIP1 c.748G > A (p.E250K) mutation (see Fig. 4 for gene mutation profile), hence, the diagnosis of PAMI syndrome was made. After administration of infliximab 5 mg/kg twice, the patient developed an allergic reaction. So we discontinued inflicimab, and started oral colchicine 0.125 mg for 1 month, which was ineffective. Therefore, we changed to subcutaneous injection of etanercept (0.8 mg/kg) once a week and methylprednisolone 1 mg/kg orally once a day. At 3-year follow-up, the patient did not have arthralgia any more and splenomegaly improved than before. The WBC remained low, between 2 and 3 × 10^9^/L. The haematocrit and platelets were normal. In addition, the CRP and AESR returned to normal. Six months before, when methylprednisolone was reduced from 4 mg to 2 mg every other day, the patient developed pyoderma gangrenosum (PG: Figs. 5 and 6), and the secretion culture showed *Enterobacter cloacae*. Besides, serum zinc level was 6.65 mg/L and faecal calprotectin level was 72.4 μg/L. we did local debridement and gave her antibiotic. In addition, we increased methylprednisolone to 6 mg/d. after treatment, her PG improved considerably (Figs. 5 and 6). The patient’s height was 78 cm and weight was 9 kg, which were 3% below the average of her age and gender before treatment. However, after treatment her height and weight were 128 cm and 31.5 kg, respectively, which were 50% for her age and gender.

### Patient 3

**Fig. 7:**
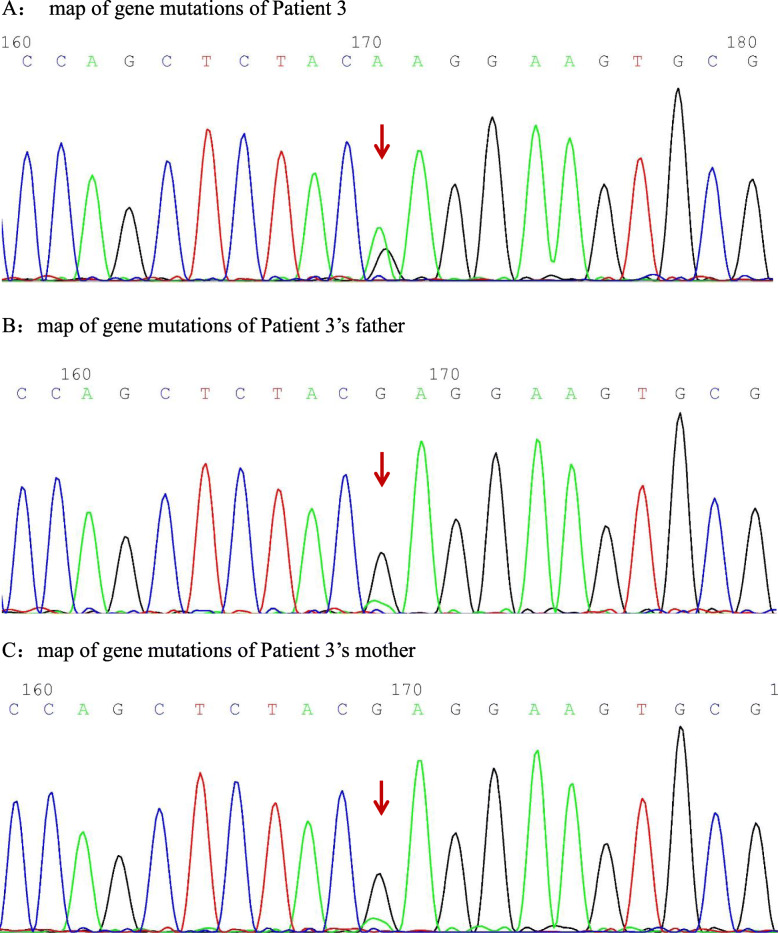
**A**: map of gene mutations of Patient 3. **B**:map of gene mutations of Patient 3’s father. **C**:map of gene mutations of Patient 3’s mother

Ten years ago, a 13-year-old male patient underwent splenectomy for trilineage hypoplasia and progressive enlargement of liver and spleen. His postoperative blood monitoring was generally normal. Two and a half years later, the child developed decreased activity tolerance and an enlarged heart. After another 2 years, the patient had fever and right knee swelling and pain. The following blood routine results were: WBC, 7.83 × 10^9^/L; neutrophil, 0.69 × 10^9^/L; Hb, 98 g/L; RBC, 3.94× 10^12^/L; reticulocyte minimum, 0.014; PLT, 883 × 10^9^/L; CRP, 139 g/L; AESR 67 mm/60 min; PCT, 3.02 ng/ml; RF, 28.3 U/ml; C3, 0.77 g/L; C4, 0.109 g/L; antiglobulin test (Coombs), ++; ANA, 1:100, and β2 glycoprotein I antibody, positive. Humoral immunity showed: IgG, 27.6 g/L; IgA, 8.18 g/L; and IgM, 1.77 g/L, indicating normal humoral immunity. Echocardiography showed: estimated sPAP, 95 mmHg, severe pulmonary hypertension, right heart enlargement and failure. Contrast-enhanced MRI of the knee showed marked thickening of synovial membrane of right knee joint and effusion. Bone marrow aspiration was normal. Aniracetam 2.5 mg qd and tadalafil 10 mg qd were prescribed as targeted therapy for pulmonary hypertension and symptomatic cardiotonic diuresis. However, her condition did not improve considerably. Whole-exome genetic testing was performed, and the results suggested a spontaneous PSTPIP1 c.748G > A (p.E250K) (see Fig. 7 for gene mutation profile) mutation. Therefore, the diagnosis of PAMI syndrome was established. The patient was prescribed oral methylprednisolone 0.5 mg/kg every two weeks, followed by oral methotrexate 10 mg after four weeks, and intravenous infliximab 6 mg/kg after eight weeks, totally 4 times. The initial treatment of methylprednisolone was 20 mg once a day. Then the dose was reduced by 4 mg per month until 8 mg QD, following by reduction of 2 mg per month. Infliximab had been used regularly throughout the entire treatment. His joint symptoms disappeared at 2-year follow-up and the hepatomegaly improved remarkably. The pulmonary hypertension disappeared. In addition, Coombs test was negative and NT-proBNP was normal, whereas CRP and ESR were 57.2 g/L, and 26 mm/60 min, respectively.

## Discussion

PAMI syndrome has been defined as distinct autoinflammatory disorder with clinical and biochemical features not found in patients with classical PAPA syndrome [1]. In addition to prominent skin inflammation and arthralgia/arthritis, PAMI is characterised by severe chronic systemic inflammation, hepatosplenomegaly, pancytopenia, and listlessness. Severe course and early onset of disease, hepatosplenomegaly, failure to thrive, cytopenia, hyperzincemia, and extremely high levels of pro-inflammatory alarmins MRP8/14 separates PAMI syndrome from PAPA. The mutations of p.E250K and p.E257K result in charge reversal in the y-domain of PSTPIP1 (E → K) and increased interaction with pyrin compared to p.E250Q mutants. Steriods, immunosuppressants, and biologics have all been reported to be effective for this disease. Clinical reports of this disease have increased recently with the widespread use of whole-exome genetic testing [2].

PSTPIP1 is a proline-serine-threonine phosphatase interacting protein, which is a tyrosine phosphorylated protein involved in cytoskeleton organisation that regulates T lymphocyte activation, leukocyte activation, cytoskeletal organisation, and interleukin-1 release. PSTPIP1 mutations are associated with periodic inflammatory flares reflected by systemic symptoms like fever or an acute phase response and local inflammation of the skin, joints, or other internal organs. At present, 25 sequence variants have been reported for PSTPIP1 gene [3]. The pathophysiological mechanisms of PAMI syndrome are not fully understood, and specific mutations in PSTPIP1 (p.E250K and p.E257K) are thought to be caused by significant increase in the binding of immunomodulatory protein pyrin because of the charge reversal of the ɣ domain. Pyrin has been reported to form an alternative inflammasome, and mutations in pyrin may lead to an uncontrolled activation of this pathway, resulting in an overwhelming production of active IL-1β. In a cellular model, it has been shown that this process can be triggered by the interaction of PSTPIP1 with pyrin. Such PSTPIP1–pyrin interaction leads to a massive release of the pro-inflammatory complex, MRPs, to form a positive feedback with IL-1, resulting in further exacerbation of the autoinflammatory process.

MRP, also known as calprotectin, is a member of the S100 protein family. S100A8 (MRP8) and S100A9 (MRP14) form a stable heterodimeric complex and are spatially extended by their C-terminal alpha-helices, which bind as non-covalent bonds to form tetramers. MRP-8/14 is mainly produced in neutrophils, monocytes, macrophages, dendritic cells, and vascular smooth muscle cells. Functionally, the subunit interface of MRP-8/14 has two affinity binding sites for bivalent zinc ions. This property of chelating serum zinc can lead to the development of hyperzincemia, giving it essential functions such as immunomodulation, resistance to bacterial infection, promotion of inflammation, and inhibition of inflammation.

Most patients with PAMI syndrome develop pancytopenia, especially neutropenia; however, the severity varies, and the aetiology is unclear. MRPs have been associated with the induction of apoptosis, inhibition of cell proliferation, and neutrophil adhesion. Therefore, it is hypothesised that MRPs are related to pancytopenia. Patients with PAMI syndrome have been reported to have severe granulocyte maturation disorder, and the reduction in granulocytes is related to anti-neutrophil antibodies. However, more cases of patients with PAMI syndrome have been reported to be negative for anti-neutrophil antibodies. Children with PAMI syndrome tend to have splenomegaly, which is considered a possible cause of pancytopenia.

The prominent cutaneous manifestation of PAMI syndrome is pyoderma gangraenosum (PG), an inflammatory skin disease with histological changes of neutrophil infiltration. Typical clinical manifestations are single or multiple skin ulcers with depressions and raised erythematous areas with violet margins, as well as pustules, bullae, abscesses, papules, nodules, and ulcers (polymorphic skin lesions, including pustules, bullae, abscesses, papules, nodules, and ulcers are characterised histologically by a neutrophil-rich inflammatory infiltrate). PG can appear in monogenic autoinflammatory diseases, namely syndromic PG, and can also be present in isolated diseases or be associated with systemic diseases, such as inflammatory bowel disease, rheumatic disease, lymphoproliferative disease, and other blood diseases.

The pyogenic arthritis of PAMI syndrome is reported to be painful and presents as recurrent aseptic monoarticular arthritis with a neutrophil-rich infiltrate, which generally occurs in childhood and may also be the first manifestation of the disease, possibly leading to joint erosion and destruction.

Compared to most autoinflammatory diseases, PAMI syndrome primarily demonstrates an increase in serum inflammatory markers, such as CRP, erythrocyte sedimentation rate, and ferritin.

Patient 1 presented with recurrent trilineage hypoplasia, splenomegaly, PG, and Ludwig’s angina. Patient 2 presented with recurrent trilineage hypoplasia, splenomegaly, pyogenic arthritis, and growth retardation. Patient 3 presented with intermittent fever, recurrent trilineage hypoplasia, hepatosplenomegaly, pyogenic arthritis, and pulmonary hypertension. In line with the literature, all three patients had recurrent trilineage hypoplasia and splenic manifestations, and the bone marrow aspiration results were negative for haematologic malignancies. Two patients had PG and pyogenic arthritis. Ludwig’s angina was present in patient 1. Pulmonary hypertension was present in patient 3, which were not reported before. In patient 3, the trilineage hypoplasia recovered completely after splenectomy, which verified the hypothesis reported suggesting that the trilineage hypoplasia was associated with hypersplenism. All three patients had significantly elevated CRP levels, and patients 2 and 3 had faster AESR, all of which agreed with inflammatory disease. All patients were positive in Coombs test, patient 1 had positive c-ANCA, and patient 3 had positive ANA and β2GP I with reduced complement C3 levels, which indicated autoimmune disease. All three children had spontaneous mutations, which were wild-type, with no PAMI syndrome-related phenotype.

The common clinical manifestations of PAPA syndrome are pyogenic arthritis, PG, acne, and anaemia [4, 5]. These clinical phenotypes overlap with PAMI syndrome. However, the prominent manifestation of PAMI syndrome is pancytopenia. Because a decrease in white blood cells and platelets has not been found in patients with PAPA syndrome. These clinical manifestations can be used as important factors to distinguish these two diseases. The currently identified pathogenic mutations in PAPA syndrome include p.A230T, p.E250Q, p.E256G, p.D246N, and p.D266N, whereas those in PAMI syndrome are p.E250K and p.E257K. Furthermore, PSTPIP1-associated inflammatory diseases also demonstrate pyogenic arthritis, PG, acne, and hidradenitis suppurativa syndrome (PAPASH) [6], pyogenic arthritis, PG, and acne-like syndrome (PAPA-like) [7], as well as PG, acne, and ulcerative colitis syndrome (PAC) [8]. The PAPASH pathogenic mutation is p.E277D, the PAPA-like pathogenic mutation is p.G258A, and the PAC pathogenic mutation is p.G403R. Hence, the above disease spectrum can exhibit a pyogenic arthritis, PG, and acne. The specific clinical characteristics such as neutropenia with hepatosplenomegaly, hidradenitis suppurativa, and ulcerative colitis can help clinicians to diagnose whether the patient has PSTPIP1-related autoinflammatory diseases, and further genetic testing is needed to clarify the type of disease [9].

There is no standard treatment regimen for PAMI syndrome; treatment with non-steroidal anti-inflammatory drugs, calcineurin inhibitors, and streoid has been reported. IL-1 antagonists, which are effective in PAPA syndrome that is also caused by mutations in the PSTPIP1 gene, have an unclear therapeutic effect in PAMI syndrome [10]. Among the three children, patient 1 was treated with methylprednisolone and cyclosporine. His rash, trilineage hypoplasia, and hepatosplenomegaly improved considerably, with only mild neutropenia and granulocyte reduction (approximately 1.5 × 10^9^/L). Patients 2 and 3 were treated with methylprednisolone and tumour necrosis factor antagonists (patient 2 was allergic to infliximab; hence, etanercept was administered). for patient 2, trilineage was improved; the inflammatory index was lower and the liver and spleen retracted. Moreover, the signs and symptoms of arthritis disappeared. Pulmonary hypertension in patient 3 returned to normal. Of the three children, only patient 1 was treated with cyclosporine and had the best effect for leukocytes and neutrophils, thus demonstrating the remarkable effect of cyclosporine on the haematological system. Tumour necrosis factor antagonists had a significant effect on the recovery of hepatosplenomegaly and joint inflammation. Steroid have contributed to the improvement of PG and the haematological system. By Searching the Literature, there was a recent report about hematopoietic stem cell transplantation (HSCT) as a therapeutic option for PAMI syndrome [11]. Five patients with PAMI syndrome underwent allogeneic HSCT with myeloablative or reduced-intensity conditioning regimens. All 5 patients engrafted; however, 1 patient at day + 13 developed hemophagocytic syndrome, followed by graft rejection at day + 17. After 5.5 months, a second HSCT was performed from an alternative donor. Another patient at day + 116 developed an intense inflammatory syndrome with significant serositis and severe mitral and aortic valve regurgitation, controlled with adalimumab, tacrolimus, and prednisone. At the last follow-up, all 5 patients have predominantly or complete donor chimerism and adequate immune recovery and are free of any PAMI symptoms. Allogeneic HSCT seems to be an effective option to cure cytopenia and severe autoinflammation in PAMI syndrome. Nevertheless, as the number of cases is small and the severity of the disease varies, the response to treatment varies. Thus, more cases of PSTPIP1-specific mutations (p.E250K and p.E257K) need to be studied to elucidate the pathophysiology and treatment strategies for PAMI syndrome.

## Conclusion

In summary, PAMI is a monogenic autoinflammatory disease caused by genetic mutations. It can clinically manifest as pyogenic arthritis, PG, acne, trilineage hypoplasia, hepatosplenomegaly, and growth retardation, as well as autoimmune diseases. Storid and immunosuppressive therapy is partially effective and cytokine antagonists can be used in refractory cases. Leukopenia is the most severe manifestation and difficult to treat for our 3 cases. Steroid play an important role in the treatment of PAMI. Cyclosporine may be effective in the treatment of leukopenia. Patients with pyogenic arthritis with early age of onset, recurrent trilineage hypoplasia, and associated skin lesions should undergo whole-exome genetic testing to achieve early diagnosis and precise treatment.

## Data Availability

Upon request.

## Abbreviations

PAMI: PSTPIP1-associated myeloid-related proteinemia inflammatory
MRP: myeloid-related protein
CRP: C-reactive protein
ESR: erythrocyte sedimentation rate
ANA: antinuclear antibodies
c-ANCA: C-antineutrophilic cytoplasmic antibodies
RF: rheumatoid factor
WBC: white blood cell
Hb: haemoglobin
RBC: red blood cell
PLT: platelet
PG: pyoderma gangraenosum
PAPA: pyogenic arthritis, pyoderma gangraenosum, acne, and anaemia
PAPASH: pyogenic arthritis, pyoderma gangraenosum, acne, and hidradenitis suppurativa
PAPA-like: pyogenic arthritis, pyoderma gangraenosum, and acne-like
PAC: pyoderma gangraenosum, acne, and ulcerative colitis
HSCT: hematopoietic stem cellt transplantation
